# Crosstalk between immune checkpoint and DNA damage response inhibitors for radiosensitization of tumors

**DOI:** 10.1007/s00066-023-02103-8

**Published:** 2023-07-07

**Authors:** Sandra Classen, Cordula Petersen, Kerstin Borgmann

**Affiliations:** 1 Laboratory of Radiobiology and Radiation Oncology, Department of Radiotherapy and Radiation Oncology, Center of Oncology, University Medical Center Hamburg-Eppendorf, 20246 Hamburg, Germany; 2 Department of Radiotherapy and Radiation Oncology, University Medical Center Hamburg-Eppendorf, 20246 Hamburg, Germany

**Keywords:** DNA repair, DNA damage response, cGAS/STING activation, Cytosolic dsDNA, PARP inhibition

## Abstract

**Purpose:**

This review article is intended to provide a perspective overview of potential strategies to overcome radiation resistance of tumors through the combined use of immune checkpoint and DNA repair inhibitors.

**Methods:**

A literature search was conducted in PubMed using the terms (“DNA repair* and DNA damage response* and intracellular immune response* and immune checkpoint inhibition* and radio*”) until January 31, 2023. Articles were manually selected based on their relevance to the topics analyzed.

**Results:**

Modern radiotherapy offers a wide range of options for tumor treatment. Radiation-resistant subpopulations of the tumor pose a particular challenge for complete cure. This is due to the enhanced activation of molecular defense mechanisms that prevent cell death because of DNA damage. Novel approaches to enhance tumor cure are provided by immune checkpoint inhibitors, but their effectiveness, especially in tumors without increased mutational burden, also remains limited. Combining inhibitors of both immune checkpoints and DNA damage response with radiation may be an attractive option to augment existing therapies and is the subject of the data summarized here.

**Conclusion:**

The combination of tested inhibitors of DNA damage and immune responses in preclinical models opens additional attractive options for the radiosensitization of tumors and represents a promising application for future therapeutic approaches.

## Introduction

Radiotherapy (RT) is one of the main therapeutic options in most cancer entities, with approximately 50% of cancer patients receiving RT as part of their cancer care [1]. Intrinsic or adapted radioresistance is therefore a tremendous challenge, because such a large proportion of cancer patients is actively treated by RT. A deeper understanding of how radioresistance occurs and the development of new strategies to overcome radioresistance is therefore an urging field of research.

The discovery of immune checkpoint inhibitors (ICIs) has improved the therapeutic options for many cancer patients across multiple tumor entities. The two most targeted structures for immunotherapy are cytotoxic T‑lymphocyte antigen‑4 (CTLA-4) and programmed cell death 1/ligand 1 (PD-1/PD-L1). The first U.S. Food and Drug Administration (FDA) approval of a CTLA‑4 antibody (ipilimumab) in 2011 was followed by the approval of two PD‑1 antibodies (pembrolizumab and nivolumab) in 2014, all exclusively for treatment of advanced-stage melanoma [2–5]. Since then, the application of ICIs has been greatly expanded to more than 15 tumor entities. Although the response rate to immunotherapy was between 20 and 50% in initial clinical trials, only around 15–20% of patients are expected to respond to immunotherapy in the long term [6, 7]. The response to ICI treatment is largely dependent on the immunological state of the tumor. Currently, it is believed that especially tumors with a high tumor mutational burden (TMB), PD-L1 expression, or high infiltration of tumor-infiltrating lymphocytes (TILs)—and thus designated as immunologically “hot”—will benefit from immunotherapy [8]. Like most drugs, the emergence of resistance, initially or adaptive, is a widely investigated challenge in the treatment with ICIs. As a result, research is moving toward combination treatments. Recently, a combination treatment of patients with ICIs and DNA damage response (DDR) inhibitors together with RT has been proposed [9, 10].

In addition to initiating immune-related processes at the local tumor, the combination of RT with ICI can also enhance the so-called abscopal effect of RT. This phenomenon is observed when one tumor site is irradiated but distant tumor sites also show a response despite no ionizing radiation (IR) being directly applied. It is believed that immune signaling mediates this effect [11] and that it might be enhanced by combined ICI treatment, as this phenomenon is extremely rare [12, 13]. The interplay between these currently developing therapeutic options is therefore the focus of the present review.

## The intrinsic cancer cell immune response

The immune response in the context of cancer is a complex, multidimensional network. The starting point for the immune response of the tumor microenvironment is DNA damage in the tumor cell. DNA damage is increasingly triggered by combination therapies including radiation, activates the DDR, and can be clinically exploited by enhancing the immune response of the tumor microenvironment. The resulting increased DNA damage leads to accumulation of cytosolic double-strand DNA (dsDNA), thereby activating the intracellular immune response via the cyclic GMP–AMP synthase (cGAS)–stimulator of interferon genes (STING) pathway. This is part of the innate immune system, which is the immediate response after pathogen-associated molecular patterns (PAMPs) or danger-associated molecular patterns (DAMPs) detection. In contrast to the adaptive immune system, it is a general mechanism that triggers unspecific inflammatory responses and does not require pathogen-specific antigens. As a result, inflammatory signaling gets activated, leading to recruitment of immune effector cells like dendritic cells (DCs), natural killer (NK) cells, and CD8+ T cells [14]. This immunogenic change in the tumor microenvironment can have positive impact, as immunologically hot tumors might be responsive to ICIs [15]. However, also immunosuppressive and tumorigenesis-favoring effects, including promotion of metastasis formation, have been observed [16, 17] and discussed detailed in Sect. “Immune signaling in response to ionizing radiation—drawbacks.” In this review the focus is on the intrinsic cancer cell immune signaling and how the interconnection of the involved pathways can be elucidated for novel therapeutic options.

### The cGAS/STING pathway

**Fig. 1:**
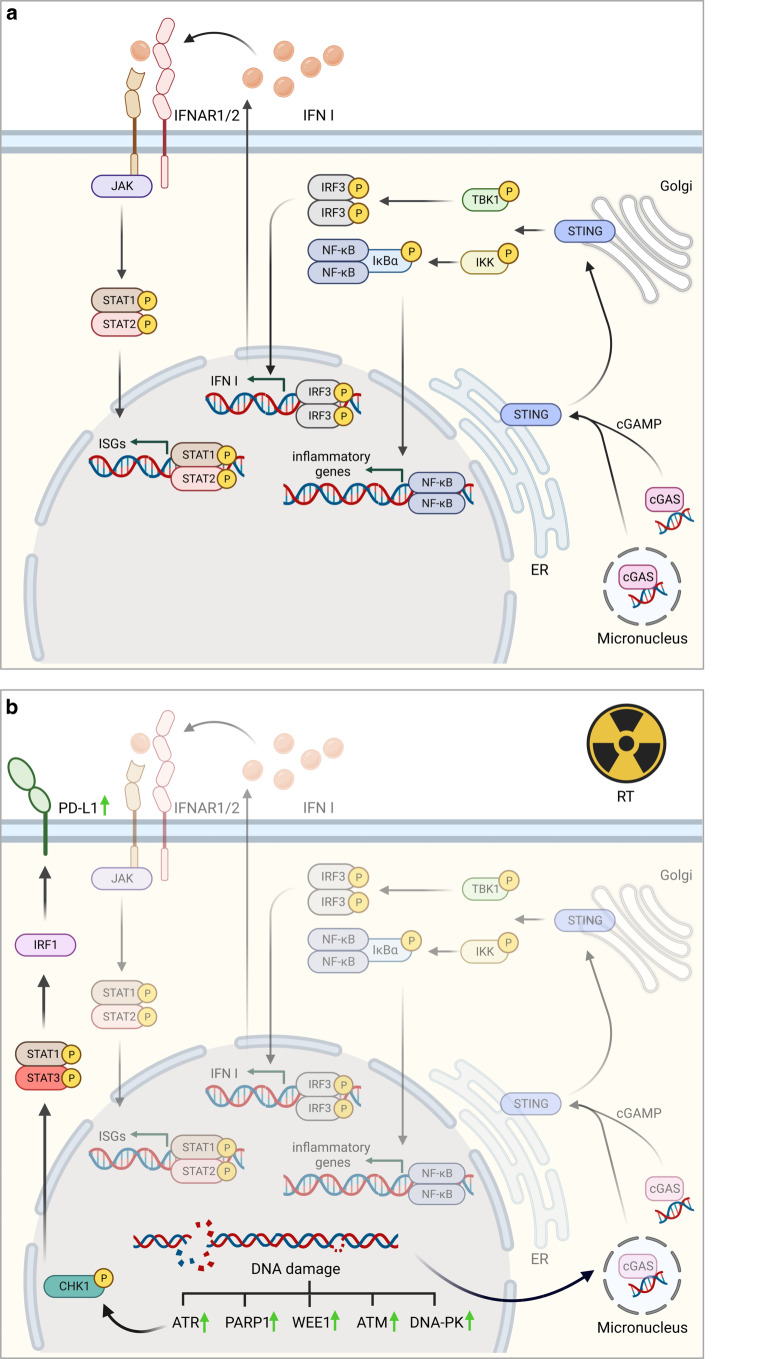
cGAS/STING activation by radiation-induced DNA damage. **a** Cytosolic dsDNA migrates in micronuclei or directly in the cytosol and is recognized by cGAS which catalyzes cGAMP synthesis after dsDNA binding. cGAMP binds to STING, which translocates from the ER to the Golgi, recruits IKK and TBK1 and activates IRF3 and IкBα, resulting in release of NF-кB. IRF3 and NF-кB translocate into the nucleus and induce transcription of IFN type I and other proinflammatory chemokines. IFN binds to IFNAR1/2 receptors, activates JAK, and STAT1/2 is phosphorylated. The heterodimer translocates to the nucleus and induces expression of ISGs. **b** Activation of DDR kinases ATR, WEE1, ATM, and DNA-PK PARP1 after IR induces DNA repair. Accumulation of cytosolic dsDNA and formation of micronuclei leads to activation of overall cGAS/STING metabolism. ATR activation affects PD-L1 surface expression via CHK1 and STAT1/3-IRF1 signaling. *ATM* ataxia-telangiectasia mutated protein, *ATR* ataxia-telangiectasia and Rad3-related protein, *cGAMP* cyclic GMP-AMP, *cGAS* cyclic GMP-AMP synthase, *CHK1* checkpoint kinase 1, *DDR* DNA damage response, *DNA-PK* DNA-dependent protein kinase, *ER* endoplasmic reticulum, *IFN* interferon, *IFNAR1/2* interferon receptor 1/2, *IKK* IκB kinases, *IRF1/3* interferon regulatory factor 1/3, *ISG* interferon-stimulated gene, *JAK* Janus kinase, *NF-κB* nuclear factor kappa-light-chain-enhancer of activated B cells, *PARP1* poly-ADP-ribose polymerase 1, *PD-L1* programmed death ligand 1, *STAT1/2/3* signal transducer and activator of transcription 1/2/3, *STING* stimulator of interferon genes, *TBK1* TANK binding kinase 1; *Wee1* Wee1 G2 checkpoint kinase. Adapted from “Blank Pathway (Linear),” by BioRender.com (2023)

One of the intracellular immune signaling cascades that is activated by cytosolic DNA is the cGAS/STING pathway (Fig. 1). This mechanism was first described in response to and for defense against pathogenic infections by bacteria or viruses [18]. It is believed to be the main activated pathway in response to cytosolic DNA. Other different pathways have been shown to be involved in PAMP and DAMP detection, including the Toll-like receptors (TLRs), retinoic acid-inducible gene I (RIG-1), and mitochondrial antiviral-signaling protein (MAVS). Since recognition involves detection of free dsDNA in the cytosol, the cGAS/STING pathway can also be triggered by the cell’s own DNA. Cytosolic DNA arises from multiple sources, including pathogens, micronuclei, and mitochondrial DNA [19]. Binding of dsDNA to cGAS induces conformational changes in the protein, activating its catalytically active site to synthesize cyclic GMP-AMP (cGAMP) from ATP and GTP [20, 21]. Recently, it was shown that the function of cGAS signaling depends on the length of the recognized DNA fragments [22]. The second messenger cGAMP activates STING upon binding, leading to STING translocation from the endoplasmic reticulum (ER) membrane to the Golgi. In its new location, STING recruits TANK-binding kinase 1 (TBK1) and IкB kinases (IKK) [23], and thereby activates interferon regulatory factor 3 (IRF3) and nuclear factor kappa-light-chain-enhancer of activated B cells (NF-кB) release by phosphorylation of IкBα [24, 25]. IRF3 and NF-кB translocate into the nucleus, where they activate the expression of type I interferons (IFNs), which in turn bind autocrine to IFN receptor 1 and 2 (IFNAR1/2), leading to activation of the Janus kinase (JAK) and signal transducer and activator of transcription (STAT) signaling pathway [26]. The phosphorylated STAT1/2 heterodimer translocates into the nucleus and activates the expression of IFN-stimulated genes (ISGs), further driving the innate immune response and connecting it to the adaptive immune response [27]. IFN signaling further leads to recruitment and maturation of DCs, which then prime CD8+ T cells for tumor infiltration [28, 29].

### Immune signaling in response to ionizing radiation—benefits

Upon DNA damage induction by ionizing radiation (IR), the integrity of the DNA is severely disrupted, resulting in genomic instability, including formation of micronuclei. Direct leakage of DNA out of the nucleus or micronuclei formation subsequently activates cGAS/STING signaling [30, 31]. Release of IFN‑1 as a consequence of cGAS/STING activation is the main driver of the antitumor immune signaling induced by IR [32] and enhances the attraction of DCs which process the tumor-associated antigens [33]. Upon maturation and migration to the lymph nodes, the DCs present those tumor antigens via the major histocompatibility complex (MHC) to the CD8+ T cells. This then allows for CD8+ T cells to infiltrate the tumor and recognize tumor-specific cells and potentially eradicate them [34]. Gupta et al. showed that RT specifically enhances tumor-specific CD8+ T cell activation by DCs, indicating how RT contributes to activation of the antitumor immune response [35]. Additionally, tumor-specific CD8+ T cell infiltration is enhanced by the release of chemokines due to activated ISG expression in response to JAK signaling. Two of the main inflammatory chemokines responsible for T cell attraction are C‑X‑C motif chemokine ligand 10 (CXCL10) and C‑C motif chemokine ligand 5 (CCL5) [36].

The cGAS/STING pathway has been suggested to also play an important role in DC activation internally. After recruitment of DCs to the immunogenic tumor site, the cGAS/STING pathway can be triggered inside the DCs, further enhancing antitumor IFN-1 signaling [37]. Different mechanisms have been proposed for how activation of the cGAS/STING pathway in non-tumor cells is mediated after IR: (i) phagosomal escape due to alkalinization of phagosomes in DCs [38]; (ii) transfer of the second messenger cGAMP from tumor cells to DCs [39], possibly via gap junctions [40]; (iii) direct exosomal transfer of tumor cell dsDNA to DCs [28]; (iv) uptake of oxidized tumor mitochondrial DNA [41].

### Immune signaling in response to ionizing radiation—drawbacks

Radiotherapy induces not only beneficial antitumor effects of the immune system but can also support immune escape mechanisms [42]. Experiments in mice showed that induction of IFN-1 signaling was dependent on the fractionation and dose of RT. Single high-dose radiation did not show the same response and abscopal effect after combined RT and anti-CTLA‑4 treatment as fractionated RT with anti-CTLA‑4 antibodies [43]. This indicates that treatment planning for combination therapies has to be designed carefully. A dose dependence was also demonstrated by Vanpouille-Box and colleagues in 2017, where a single dose of 20 Gy upregulated three prime repair exonuclease 1 (TREX1) expression, while fractionation of 3 × 8 Gy upregulated ISG expression in patient-derived xenografts of a transcription factor p53 (TP53)- and GTPase K‑Ras (KRAS)-mutated lung adenocarcinoma [44]. TREX1 is a cytoplasmic DNA exonuclease which degrades the dsDNA arising in the cytoplasm due to IR; thus, high expression would suppress cGAS/STING activation, inhibiting the intracellular antitumor immune signaling cascade [44]. The same group later showed that TREX1 can also be transmitted via exosomal shuttling, inhibiting cGAS/STING activation in DCs [28]. However, this mechanism appears to be dependent on the cell system studied. Other studies have shown that tumors, which suppress cGAS/STING signaling due to mutations or epigenetic silencing, can also escape induction of the IFN-1 response [45, 46].

Chronic activation of IFN-1 signaling in a tumor can result in immunosuppressive behavior [47]. In the study of Benci et al., the authors showed that continuous IFN signaling was associated with STAT1-related epigenomic and transcriptomic modifications, which lead to PD-L1-independent adaptive resistance [47]. Another study from Bakhoum et al. in 2018 showed that chromosomal instability in tumor cells can lead to activation of the cGAS/STING pathway. This activation was associated with metastasis formation driven by the noncanonical NF-кB signaling pathway [16]. The same pathway was shown to be activated in DCs and negatively affect IR-induced IFN-1 signaling. It was further shown that inhibition of the noncanonical NF-кB pathway led to tumor regression by treatment with IR [48].

However, these identified adverse effects of RT for the immune response provided deeper insights into how it may be possible to overcome nonresponsive phenotypes or acquired resistances.

## Genomic instability as a predictive biomarker for immune therapies

Immunotherapy is currently transforming existing tumor treatment. ICIs have achieved tremendous therapeutic success in numerous tumor types, including cancers traditionally considered nonimmunogenic. However, a significant proportion of patients do not respond to these therapies. Therefore, early selection of susceptible patients is critical, and the development of predictive biomarkers is one of the major challenges in ICI development. Starting from the immunogenic side, the expression of PD-L1 and infiltrating T lymphocytes are the most frequently used biomarkers of potential response to tumor therapy [49]. Recent publications suggest that the genomic landscape of the tumor, its mutational burden, and tumor-specific neoantigens are potential determinants of the response to ICI and may therefore influence immunotherapy outcomes. In addition, tumor-associated defects in DNA repair mechanisms have been associated with improved survival and durable clinical benefit from ICI. Thus, the TMB and associated tumor-specific neoantigens appear to be important predictive approaches to anticipate potential clinical benefits of ICI, as they closely reflect the repair capacity of tumor cells and their intrinsic genomic instability. Initially, studies showed that TMB is correlated with the clinical benefit of anti-PD‑1 and anti-CTLA‑4 therapy in several tumor types, including malignant myeloma, non-small cell lung cancer (NSCLC), and various tumors with DNA repair deficits [50]. Overall, a direct correlation between DNA repair deficiency, TMB, predicted neoantigen load, and clinical activity of ICI is suggested.

### Tumor mutational burden

A correlation between TMB and response to ICI has been enabled by recent advances in next-generation sequencing (NGS) technology, particularly in whole-exome sequencing and RNA sequencing. High mutation load, defined as > 100 nonsynonymous single-nucleotide variants (nsSNV) per exome, was associated with clinical benefit in melanoma patients treated with anti-CTLA‑4 therapy [51, 52]. Rizvi and colleagues correlated high TMB (defined as > 178 nsSNVs per exome) and durable clinical benefit in two independent cohorts of NSCLC patients receiving pembrolizumab [53]. Of note, the study reported a significantly increased overall response rate (ORR) in tumors with a smoking-related molecular signature, which are potential determinants of response to ICI. In addition, mutations in DNA repair genes, including DNA polymerase delta 1, DNA polymerase epsilon catalytic subunit, mismatch repair gene MutS homolog 2 (*MSH2*), breast cancer gene 2 (*BRCA2*), RAD51 gene homolog C (*RAD51C*), and RAD17 checkpoint clamp loader compound (*RAD17*) were observed in responders with the highest mutational burden. This supports the notion that DNA repair defects may increase tumor immunogenicity by promoting somatic mutations. Consistently, later findings showed higher response rates to anti-PD‑1 therapy in mismatch repair (MMR)-deficient tumors and in *BRCA2*-mutated melanoma [50].

Major challenges that remain to be addressed to improve the robustness of TMB include the definition of optimal tumor purity and sequencing depth, as well as the threshold for defining “high” and “low” mutation burden. Indeed, there is a significant overlap in the mutational range between responders and nonresponders [51, 52]. Some patients still benefit from ICI despite very low mutation rates, and conversely, high TMB does not always correlate with response. This is best illustrated in relapsed or refractory Hodgkin’s lymphoma, which is very sensitive to PD‑1 blockade despite having virtually no mutation [54]. Mutational signatures, which are functional slices of past and current disease biology related to DNA damage and DNA repair, may provide an additional genomic determinant of response to ICI. Their use, combined with assessment of TMB and detection of mutations in DNA repair genes, may therefore allow better grouping of patients and identify ICI-sensitive tumors. Importantly, the mutational landscape analyses described above provide only an *instantaneous and descriptive* picture of a tumor genome. Mutational signatures may even in some cases exclusively reflect past DNA repair deficiencies and may not be relevant markers of the current DNA repair status of the tumor. It is therefore critical to evaluate the potential of these mutations to functionally enhance the antitumor immune response through the generation of immunogenic neoantigens.

## Link between DNA repair and the immune response

Understanding the mechanism underlying DNA damage-derived signal transduction is critical for overcoming refractory cancer, especially when cancer immunotherapy is used in combination with DNA damage-dependent radio-/chemotherapy. Several lines of evidence suggest a link between DNA damage signaling and modulation of the immune response. In this process, DNA damage-triggered signals within the cell of origin are transmitted to the cell surface and neighboring cells, modulating immune and inflammatory responses. The interplay between PD-L1 expression, microsatellite instability (MSI), and accumulation of mutations in the cancer genome leads to production of neoantigens and presentation of the human leukocyte antigen (HLA)–neoantigen complex in cancer cells. HLA neoantigen presentation promotes immune activity in the tumor environment and characterizes a so-called hot tumor [55]. Several studies have shown that PD-L1 expression is upregulated in cancer cells in response to increased DNA damage [56–60]. This may be triggered by loss of individual DNA repair proteins responsible for double strand break (DSB) repair, such as BRCA2 or Ku70/80, after irradiation [58]. Likewise, defects in other DNA repair pathways can lead to upregulation of PD-L1 [56]. The common mechanism leading to upregulation of PD-L1 occurs through activation of the DNA damage response. This directly affects activation of the STAT1/3-IRF1 signaling cascade, downstream of which PD-L1 mRNA transcription is activated [58]. Supporting this observation, it was shown that DNA repair-deficient breast tumors were associated with CD4+ and CD8+ lymphocyte infiltration and that cells from these DNA repair-deficient breast tumors expressed the chemokines CXCL10 and CCL5 more strongly [59].

In terms of RT delivery, this means that a wide range of diverse DNA damage, including base damage, single-strand breaks (SSBs), DSBs, and DNA crosslinks (ICLs), is induced [61]. DNA damage induced by IR activates the DDR, which subsequently regulates the appropriate DNA repair pathway choice. The DDR represents a complex network consisting of cell cycle control, DNA repair, and inactivation of multiple interconnected signaling pathways and mechanisms, all aiming to maintain cell viability. Once DNA damage is detected, cell cycle checkpoints are activated to arrest the cell cycle for DNA repair prior to cell division. This allows the cell to survive genomic instability and replication stress through successful DNA repair, or to initiate permanent arrest or cell death. The DDR consists of a series of pathways with different protein sets specialized for specific types of damage and classified as sensors, transducers, and effectors. Over the past few years, it has become more evident that failure or defectiveness of individual components of the DDR machinery leads to fragmentation of DNA, which enters the cytoplasm and thereby contributes to immune signaling in the tumor [31, 57, 59, 62, 63].

### Recognition of DNA damage

Double-strand breaks are the most lethal event in a cell and are therefore rapidly recognized by the MRE11-RAD50-NBS1 (MRN) complex, which interacts with chromatin and promotes activation of ataxia-telangiectasia mutated (ATM) kinase by subsequent autophosphorylation at Ser1981 [64]. The ATM kinase, discovered in 1995 by Y. Shiloh [65], is an essential sensor of DNA damage by activating hundreds of different substrates, including TP53 and checkpoint kinase 2 (CHK2) [66]. In addition, ATM enables phosphorylation of histone H2AX to form H2AX foci [67], which is essential for the repair of DSBs.

Single-strand breaks are recognized by another repair pathway, the RAD9-HUS1-RAD1 complex, which in cooperation with RAD17, replication factor C (RFC) 2, RFC3, RFC4, and RFC5 activates the ataxia-telangiectasia and Rad3-related (ATR) kinase [68]. ATR-interacting protein (ATRIP)-dependent recruitment of ATR to replication protein A (RPA)-bound single-stranded DNA is initiated upon ATR activation [69, 70], leading to phosphorylation of checkpoint kinase 1 (CHK1) [71].

CHK1 and CHK2 then further transmit the signaling by phosphorylation of various downstream effectors. CHK2 suppresses phosphatase cell division cycle 25 A (CDC25A), which abrogates suppressive phosphorylation of cyclin E/cyclin-dependent kinase (CDK) 2 and cyclin A/CDK2 complexes, preventing entry into the S‑phase of the cell cycle [66]. CHK1 regulates the G2/M checkpoint by activating G2 checkpoint kinase (WEE1), which then phosphorylates CDK1, reducing its activity and preventing entry into mitosis [72]. In addition, CHK1 modulates the S‑phase checkpoint by facilitating degradation of CDC25A phosphatase, whose activity is critical for removing the suppressive phosphate groups of CDK4 and CDK2 kinases and ensuring cell cycle progression [73].

Another important kinase responding to DNA damage is the catalytic subunit of DNA-dependent protein kinase (DNA-PKcs), one of three related kinases in the phosphatidylinositol 3‑kinase-related kinase (PIKK) family. These kinases are activated after DNA damage and phosphorylate many downstream targets to activate DNA damage checkpoints and stimulate DNA repair [74–76]. PIKKs function in overlapping DNA damage signaling networks. Regarding DSB repair, despite extensive crosstalk between PIKKs, the generally accepted view is emerging that DNA-PKcs play a direct and central role in DNA repair through nonhomologous end-joining (NHEJ), while ATM and ATR play a critical role in promoting repair through homologous recombination (HR) of open DSBs and DSBs at collapsed replication forks, respectively (summarized in [77]).

### DNA repair pathways to eliminate IR-induced DNA damage

Double-strand breaks are mainly repaired by two processes: NHEJ and HR. NHEJ joins ends without requiring a repair template; therefore, it is error prone and typically results in small deletions or insertions at repair sites. NHEJ functions throughout the entire cell cycle and is the dominant DSB repair pathway [78]. HR requires a homologous repair template and is thought to be generally error free [79–81]. HR is largely restricted to the S/G2 phases of the cell cycle and is mediated by the recombinase RAD51, which is loaded onto the DNA by interaction with the BRCA1, BRCA2, and partner and localizer of BRCA2 (PALB2) complex [82, 83]. HR is important for precise repair of open DSBs, those directly induced by IR, and is critical for repair of replication-associated DSBs, including those that arise when replication forks encounter radiation-induced single-strand lesions [84].

In addition to HR and NHEJ, a “backup” DSB repair pathway has been discovered in tumors. It has similar mechanisms to the two main DSB repair pathways but is genetically distinct and referred to as alternative end-joining (a-EJ) or microhomology-mediated end-joining [85]. The a‑EJ engages similar initiation processes and factors to HR or NHEJ regarding the connection of open DNA ends, as the MRN complex is involved in a‑EJ initiation. The polymerase theta has been shown to be essential for this pathway [86], as well as poly(ADP-ribose) polymerase 1 (PARP1) [3]. These backup repair pathways cause gene deletions, translocations, and rearrangements in cancer cells. Currently, there is increasing interest in a‑EJ signaling pathways as potential therapeutic targets due to cancer cell specificity [86, 87].

Irradiation-induced base damage and SSBs are rapidly and efficiently repaired by base excision repair (BER). X‑ray cross-complementing protein 1 is the facilitator of BER, as it interacts with DNA ligase III, polymerase β, and PARP1 [88].

Nucleotide excision repair (NER) is conducted by the cell to remove bulky DNA lesions [89]. Removal of these lesions is mediated by several different complexes, and deficiencies of involved proteins lead to severe diseases like xeroderma pigmentosa or Cockayne syndrome [90].

The MMR pathway is required to detect and repair base–base mismatches of the DNA during replication or in response to induced DNA damage. The MSH heterodimers of MSH2/MSH6, detecting base–base mismatches and small insertions or deletions, and MSH2/MSH3, detecting also larger insertions or deletions, together with PMS1, PMS2, MLH1, and MLH3 are the recognition sensors of MMR [91]. Interactions with additional proteins like proliferating-cell nuclear antigen, exonuclease 1, RPA, and RFC then cause excision of the mismatches and repair. Mutations of proteins involved in MMR have been shown to lead to MSI [91].

DNA crosslinks between and within DNA strands represent a dangerous form of damage that blocks vital cellular processes such as transcription and replication. The Fanconi anemia (FA) pathway is responsible for repairing these aberrations in the DNA structure [92, 93]. Fanconi anemia is a heterogeneous genetic disease involving 22 different genes that can be divided into three main groups: the FA core complex, the I‑D2 complex, and the downstream FA proteins [94]. The FA pathway allows for unblocking of the replication fork by inducing formation of a DSB and coordinating the action of three critical repair mechanisms: translesion synthesis bypasses the lesion, and after removal of toxic adducts by NER, the gap is closed by HR [93].

## Translational aspects of DNA repair and immune signaling connection

Several studies indicate a correlation between DNA damage and the immune response. Particularly in tumor cells with deficiencies in the DNA repair pathways HR and MMR, increased immunogenicity has been shown to correlate with response to ICIs [95, 96]. MMR deficiency, which is known to lead to MSI, has already been identified as a prognostic marker for the use of ICIs [95]. However, MSI is mainly observed in three cancer entities: endometrial, gastric, and colorectal cancer. Identification of additional DNA repair defects to predict response to ICIs is pending.

These observations further imply that targeting the DDR in combination with ICI may be a promising target for intensified therapy. Triple combinations of DDR inhibitors, ICI, and RT are currently being investigated, as RT-induced DNA damage could be even more effective in causing cancer cell death when DDR is inhibited and immune response signaling is maximized.

### Combined therapy of ICI with PARP inhibition

Mutations in the HR protein BRCA1 were associated with higher infiltration of T lymphocytes in a cGAS/STING pathway-dependent manner in breast cancer cells [59]. This was due to increased release of the chemokines CXCL10 and CCL5 upon cGAS/STING activation. The same study showed that PD-L1 expression was enhanced by DNA damage in S phase, also dependent on cGAS/STING [59]. Loss of *BRCA2* was observed to lead to increased activation of innate immune signaling. This led to chronic upregulation of ISG expression and could be enhanced by PARP1 inhibition [9]. However, it was also observed that a deficiency in BRCA1 or 2 did not lead to the same modulation of the immune response, indicating the complexity of the connection between the DDR and the immune response [97]. Inhibition of PARP1 has been shown to be effective in treatment of tumors carrying mutations in the *BRCA1* or *BRCA2* genes due to synthetic lethality. In these tumors, the accumulation of SSBs upon treatment with PARP1 inhibitors leads to blockage of replication forks and the formation of DSBs [98–100]. More recently, it has become evident that PARP1 inhibition can directly activate the cGAS/STING pathway independent of the BRCA status of the tumor [101]. PARP1 inhibition caused a time-dependent upregulation of the chemokines CXCL10 and CCL5, which was reversed in cells with knockdowns in the key proteins of the cGAS/STING pathway. These observations in cell lines were confirmed by in vivo studies, where CXCL10 and CCL5 upregulation led to increased CD8+ T cell infiltration. A combination treatment of PARP1 inhibition and ICI potentiated the therapeutic effects in colon and ovarian cancer mice models [101]. Another study investigating the effects of PARP1 inhibition on intracellular immune signaling revealed that activation of cGAS/STING is dependent on PARP1 trapping, as treatment of PARP1-deficient cells with the PARP1 inhibitor talazoparib did not show any activation of the cGAS/STING pathway [102].

In the study from Sato et al. it was shown that PD-L1 upregulation was caused in an ATM/ATR/CHK1-dependent manner [58]. Cells deficient in BRCA2 or Ku80 showed increased PD-L1 expression after IR or PARP1 inhibition, but this effect was suppressed again upon CHK1 inhibition. They further revealed that the upregulation required signaling via STAT1/3 and IRF1 [58].

### Combined therapy of ICI with DDR inhibition and radiation

Increasingly, the combination of DDR inhibitors and ICIs with radiation is being investigated in preclinical tumor models (Fig. 1). Current progress in the clinical implementation of immunostimulatory DNA-damaging treatment regimens in combination with radio-/chemotherapy and the necessary future directions to optimize the immunosensitizing potential of DNA damage response inhibitors were summarized by [103].

#### Combined therapy of ICI with ATR inhibition and radiation

Because of its exclusive importance in regulating the replication stress level, most of the available studies involve inhibition of ATR. The ATR kinase inhibitor AZD6738 was observed to attenuate radiation-induced CD8+ T cell depletion and enhance CD8+ T cell activity in mouse models of *KRAS*-mutated cancer in combination with conformal RT. Mechanistically, ATR inhibition by AZD6738 appears to block radiation-induced PD-L1 upregulation on tumor cells, thereby reducing the number of tumor-infiltrating regulatory T cells (Tregs). Of note, AZD6738 in combination with conformal RT can induce immunological memory in treatment-responsive mice [104]. Feng et al. observed that inhibitors of the DNA damage response kinase ATR can significantly enhance innate immune responses triggered by IR. They showed that both the cGAS/STING-dependent DNA-sensing pathway and the MAVS-dependent RNA-sensing pathway are responsible for type I IFN signaling induced by IR. The authors suggested that DNA fragments released due to DNA damage may either activate the cGAS/STING pathway or be transcribed, thereby initiating MAVS-dependent RNA sensing and signaling. Both observations suggest that different pathways are involved in type I IFN signaling in response to DNA damage and may thus represent a promising new combination therapy against cancer [105]. Also, an enhanced tumor-inhibitory effect of ATR inhibition in combination with fractionated RT was shown in an immunocompetent mouse model for human papillomavirus (HPV)-positive malignancies. Here, significant radiosensitization by the ATR inhibitor AZD6738 was observed, accompanied by a marked increase in DNA damage and immune cell infiltration. In parallel, increased numbers of CD3 and NK cells were observed. ATR inhibition plus IR resulted in a gene expression signature consistent with the observed type I/II IFN response. Increased MHC I levels were monitored on tumor cells, with transcriptional level data indicating increased antigen processing and presentation in the tumor. In vivo, significant modulation of cytokine gene expression (particularly *CCL2, CCL5*, and *CXCL10*) was observed. In vitro data also indicate that *CCL2*, *CCL5*, and *CXCL10* were increasingly expressed by tumor cells after ATR inhibition plus RT [106]. In an HPV-negative mouse model of oral squamous cell carcinoma, Patin et al. observed that inhibition of ATR enhanced IR-induced inflammation of the tumor microenvironment, with NK cells playing a central role in maximizing treatment efficacy. It was found that ICI can further enhance the antitumor activity of NK cells [107].

Sheng et al. also observed immune-stimulatory effects of the ATR inhibitor AZD6738 in combination with IR and ICI in hepatocellular carcinoma. It was found that AZD6738 increased IR-stimulated CD8+ T cell infiltration and reversed the immunosuppressive effect of IR on the number of Tregs in mouse xenografts. Moreover, the addition of AZD6738 enhanced infiltration; increased cell proliferation and the ability to produce IFN‑γ from CD8+ T cells derived from TILs; and caused a decreasing trend in the number of TILs and Tregs, and depleted T cells in mouse xenografts. This significantly improved the immunological microenvironment of the tumor [108].

#### Combined therapy of ICI with ATM inhibition and radiation

For inhibition of DDR signaling via ATM, it was shown that activation of the innate immune response through enhanced induction of DNA damage could increase the efficacy of ICI [109]. The inhibition of ATM alone was already able to increase tumoral type I IFN expression independently of the cGAS/STING pathway. This was done in a TBK1 and proto-oncogenic tyrosine protein kinase Src-dependent manner. The combination of ATM inhibition and IR increased TBK1 activity even more markedly and, accordingly, increased IFN production and antigen presentation. In addition, silencing of ATM increased PD-L1 expression and enhanced the sensitivity of pancreatic tumors to PD-L1-blocking antibodies. This was associated with an increase in tumor CD8+ T cells and established immune memory [109]. The authors also found that low ATM expression inversely correlated with PD-L1 expression in patients’ pancreatic tumors. Overall, these results indicate that the efficacy of ICI in pancreatic cancer is enhanced by ATM inhibition and further enhanced by IR, depending on the increased immunogenicity of the tumor [109].

#### Combined therapy of ICI with DNAPKcs inhibition and radiation

Inhibition of the DDR sensor kinase DNA-PKcs, which is responsible for NHEJ, was also demonstrated to have an immunomodulatory effect [110]. The combination of IR and DNA-PKcs inhibition was shown to enhance cytosolic dsDNA and tumor-associated type I IFN signaling independently of cGAS and STING. In parallel, PD-L1 expression was stimulated upon DNA-PKcs inhibition and IR. Simultaneous use of anti-PD-L1 in combination with IR and DNA-PKcs inhibitors potentiated antitumor immunity in pancreatic cancer models [110].

#### Combined therapy of ICI with WEE1 inhibition and radiation

As another interesting DDR protein for stimulating the immune response, WEE1 was investigated in combination with IR regarding its effects for cell killing by T lymphocytes and a sensitizing effect for ICI. In several models it was observed that the WEE1 inhibitor AZD1775 led to DNA damage accumulation and that the combination treatment improved tumor control in a syngeneic mouse model of oral cavity cancer (MOC1) in vivo. Combination treatment enhanced granzyme B‑dependent T lymphocyte killing by reversing additive G2/M cell cycle blockade. The combination of IR and AZD1775 improved CD8+ T cell-dependent control of MOC1 tumor growth and the rate of complete eradication of established tumors in the context of the PD-axis ICI. Functional assays demonstrated enhanced tumor antigen-specific immune responses in sorted T lymphocytes. The combination of IR and AZD1775 not only increased tumor-specific cytotoxicity, but also improved susceptibility to killing by T lymphocytes and response to PD-axis ICI [111].

#### Combined therapy of ICI with STING antagonists and radiation

Very recent data in a preclinical model showed that not only cell surface ligands such as PD-L1 or PD-L2 are suitable for combined therapy of ICI and DDR inhibitors for enhanced radiation sensitization. It was observed that intracellular immune response proteins like STING antagonists also resulted in significant radiation sensitization with improved survival in a syngeneic genetically engineered mouse model and human pediatric high-grade glioma cells [112]. Significantly improved survival was observed when the PARP inhibitor pamiparib was combined with the STING antagonist H151 after IR. The CHK1 inhibitor was also shown to prolong survival in a mouse model when combined with H151 and IR [112].

## Perspective

Defects in the DDR can trigger intracellular immune signaling endogenously. This effect can be enhanced by exogenously induced DNA damage via DDR inhibition, chemotherapeutics, or RT. The combination of DDR inhibitors and RT may be able to force tumors, which are not endogenously inflamed, to activate proinflammatory signaling. The intracellular immune signals are then transmitted to the tumor microenvironment by release of IFNs and chemokines, leading to recruitment of immune effector cells. This suggests that the combination of the recently tested inhibitors of DDR and immune response in preclinical models opens new options for radiation sensitization and thus represents an attractive option for promising use in cancer therapy.
